# When Veterinarians Support Canine Therapy: Bidirectional Benefits for Clinics and Therapy Programs

**DOI:** 10.3390/vetsci5010002

**Published:** 2018-01-04

**Authors:** John-Tyler Binfet, Haley J. Silas, Sean W. Longfellow, Katrina Widmaier-Waurechen

**Affiliations:** 1 Faculty of Education, University of British Columbia, Kelowna, BC V1V 1V7, Canada; 2 Faculty of Arts & Sciences, University of British Columbia, Kelowna, BC V1V 1V7, Canada; hsilas84@gmail.com (H.J.S.); longfellowsean@gmail.com (S.W.L.); katrinawidmaier@gmail.com (K.W.-W.)

**Keywords:** canine therapy, client recruitment, community collaboration

## Abstract

This paper proposes a mutually beneficial model of collaboration between veterinarians and canine therapy programs. Veterinarians and the clinics for whom they work routinely establish collaborations with multiple and varied stakeholders. This might include a laboratory for processing samples and the corresponding courier company needed to deliver samples to the lab or a partnership with a local dog rescue organization for whom discounted rates are offered. One community partnership that stands to benefit both the clinic and the community agency, is for veterinarians to work in tandem with a local canine-assisted therapy program. The benefits to such an alliance are multifold and address aspects of veterinary medicine including client recruitment, community education, and access to a network of devoted dog enthusiasts.

## 1. Introduction

Veterinarians and their associated clinics routinely establish connections, collaborations, and partnerships with a variety of community stakeholders that help their practice thrive. The clinic or clinician has either a need (e.g., lab samples to process) or offers a service (e.g., the treatment of animals from a local rescue group at a discounted rate) that drives these relationships. One relationship that remains underexplored is the relationship between the veterinarian and his/her local canine therapy program. The aim of this paper is to outline a collaborative model between veterinarians and their local canine therapy program and explicate the bidirectional benefits of this collaboration to each agent.

Veterinarians establish collaborations with a variety of community stakeholders. In fact, veterinary clinics are typically situated within communities where they will be in direct contact with various clients, businesses, and organizations. Some of the partnerships between veterinarians and clients may include but are not limited to: breeders, pet owners, law enforcement, dog daycare facilities, sporting or agility groups, and training or behavioural programs. The businesses that veterinarians collaborate with are of even larger variety, including but not limited to: lab services and supplies, couriers, office supply companies, pharmaceutical companies, and food manufacturers. Finally, the organizations with whom veterinarians collaborate with may vary by region and by community, but often include organizations such as local dog rescue groups, local societies for animal welfare, wildlife organizations, animal control agencies, and volunteers and/or volunteer programs. It is important to note that the organizations with whom veterinarians collaborate with may be large-scale, or small not-for-profit organizations that are locally established, and are therefore unique to the clinician’s community.

## 2. A Model of Clinic-Organization Partnership

A common partnership found within veterinarian clinics is their relationship with local dog rescue organizations. These organizations typically take in dogs from high-kill shelters or dogs who, for a variety of reasons, are surrendered and require rehoming. Some of these rescue organizations are breed specific and thus require specialized clinical knowledge and treatment (e.g., treating brachycephalic in bull breeds). What is evident in this partnership is the bidirectional benefits to both the clinic and the rescue organization. From the clinic’s perspective, the relationship brings in a steady stream of canine patients, patients who typically receive a wellness check, vaccinations and sterilization if not yet done, and additional treatment pending their health profile. Once adopted, and providing the adoptive family is in the same geographic catchment area, the adopted dogs typically stay on as clients. As most rescue organizations rely on donations to operate, the standing benefit to rescue organizations arising from an established partnership with a veterinarian or clinic includes the negotiation of discounted clinic rates. It will be argued shortly that a similar partnership can be established between veterinarians and canine-therapy organizations.

## 3. Understanding Canine-Assisted Therapy

There has been a surge in Animal-Assisted Activities in the past decade with programs moving beyond the traditional model of one volunteer handler and canine working with a health-impaired client in a static setting [1,2]. As an illustration of this, within the context of post-secondary education alone, Crossman and Kazdin (2015) identified 925 on-campus canine therapy programs across North America [3]. The field of canine therapy is burgeoning and now sees the innovative incorporation of therapeutic canines into a variety of settings. We now routinely see therapy dogs working in elementary schools to assist reluctant readers [1,4], in courtrooms to support children giving testimony, in airports to reduce traveler stress, in funeral homes to comfort mourners, and on college campuses to support student mental health and well-being [5,6,7,8].

The popularity of therapy canines is a reflection of the public’s appetite for two distinct interactions with canines: (1) intentional or purposeful interactions with dogs; and (2) the desire for ambient interactions (i.e., just being in the presence of a dog). This may be a reflection of the extent to which dogs are considered family members [9]. Conservative current estimates indicate that, when prompted to identify their family members, participants in studies include their dog as a family member 63% of the time [10]. As an illustration of this, new research on canine weight loss reveals that weight gain is common within families and both family members and canines themselves correspondingly gain (and lose) weight [11]. As a reflection of this repositioning of dogs from the backyard to the family room, dogs are routinely incorporated into the day-to-day life of their owners (e.g., dogs are often seen attending move-in day on college campuses to bid freshmen students farewell). There is a societal desire to interact with dogs and we increasingly see the incorporation of dogs into day-to-day routines. This is evidenced by the sheer number of businesses and corporations allowing employees to bring their dogs to work [12].

One means of providing purposeful interactions with canines is through participation in a canine therapy program. Here, the client is afforded an opportunity to interact with one or more therapy dogs within a specified setting. Overseeing a canine therapy program is no small undertaking and directors of such programs bear great responsibility in ensuring that dogs used in their programs are of sound temperament and well-suited to this public work. Though interest runs high from the general public around participating in canine therapy, finding well-qualified volunteer handlers and canines can prove problematic for many agencies [6]. A well-qualified handler must demonstrate canine management skills that provide adequate support, restraint, and direction [13]. Further, assessing dog-handler teams is time consuming and finding reliable teams is a challenge for those working in the field [7]. Across programs, there are standard behavioural benchmarks used by agencies to assess therapy canines in the areas of reliability, predictability, controllability and suitability [14]. Because veterinary visits may be stressful for both the handler and his/her dog [13], this is an ideal setting to informally assess or initially screen the suitability of dog-handler teams.

It can be argued that when assessing dog-handler teams for participation in canine therapy, the emphasis has traditionally been on assessing canine disposition and behaviour, with handler qualifications underemphasized [15]. Increasingly, there is discussion around the importance of assessing handler knowledge and skill and ensuring that the handler is well-suited to the target client population [15]. Strong canine therapy programs will thoroughly assess canines but also provide training and ongoing assessment of volunteer canine handlers.

## 4. Complementary Needs

The above illustrates the primary needs of a canine therapy program—notably, to identify strong potential dog-handler teams who are able to fulfill the program’s mission through their reliable participation in sessions. Veterinarians and clinics too have needs and certainly one need driving the economic viability of clinics is client traffic. Although reports have revealed that Canadians are spending more money on their pets than ever, veterinary visits are declining [16,17]. It is proposed here that a key benefit to a collaboration between veterinarians and canine therapy programs is the bidirectional recommendation of clients. As the total number of clients per clinic can be a misleading indicator of clinic productivity (due largely to clinics maintaining inactive files), active clients (clients who have been to the practice in the past year) are the clients generating clinic revenue [18].

Although no turnover rates of dog-handler therapy teams could be found, the clients of veterinary clinics, due largely to the relatively short lifespan of animal clients, experience relatively high rates of client turnover. Urfer and colleagues (2011) reported the mean life expectancy for 72 different canine breeds. Collapsing their data across breeds, we calculated the mean life expectancy across breeds to be 12.2 years (*SD* = 1.97; range 7–16.5 years [19]). In light of this constant turnover of clients, much has been written on the importance of generating new clients to build and maintain the economic viability of small animal practices [17]. Graham-Mohl (2013) argues that, to maintain economic viability, clinics must be community-focused and tailor their marketing efforts to the needs of the local community [20]. Rowe (2007) argues that the key challenge to a clinic is, in fact, to maintain its client base [21]. Below, we argue that an under-accessed client population lies in clients who volunteer with their dog within the context of canine therapy.

## 5. Volunteer Handlers Make Ideal Clients

Within the dog owning community, therapy dog handlers are particularly invested in their dogs. This is evidenced by the sheer commitment required to: (a) be accepted into a reputable canine therapy program; and (b) the ongoing time and energy required of handlers to ensure their dog is prepared for therapeutic work (e.g., grooming, transporting to and from site visits). Canadians are spending more than ever on their pets. The annual cost of care for a dog reported by the Ontario Veterinary Medical Association is $1386 [22]. Additionally, spending associated with owning a pet is predicted to increase [23]. In 2016, pet owners in the U.S. spent $66.75 billion on their pets, with $15.95 billion spent on veterinary care [24]. It can be argued that of the pet owning population, canine therapy volunteer handlers are particularly devoted to the care and well-being of their dogs. This, in turn, translates to frequent clinic visits to ensure their dog is in optimal health. The average number of veterinary visits per household per year for dogs is 2.6 [10]. Though the rate of clinic visits by canine therapy dog handlers has not been reported, it is likely that their annual number of visits surpasses the average per dog owning household. Canine therapy dog handlers are, in many ways, the ideal client.

Extending beyond actual clinic visits, canine therapy volunteers are typically active within their local dog community. Certainly, this is evidenced by participation in social media and via informal, word-of-mouth communication typical of the communication pathways of members of micro-communities. Thus, an established relationship with a local canine therapy program provides a clinician with access to a network of dog enthusiasts—enthusiasts who are devoted to the care and well-being of their dogs and who are high consumers of veterinary services. Such a relationship stands to boost referrals well beyond the immediate realm of a given canine therapy program.

## 6. Veterinarians and Community Responsibility

As reflected in regional, national, and international codes of professional conduct, there is an expectation that veterinarians engage in different forms of community engagement and public education (see Table 1). Liaising with a local canine therapy program provides an avenue through which veterinarians may both directly and indirectly engage with local communities. Directly and through their recommendations, veterinarians may impact the quality of dog/handler teams participating in local canine therapy programs thus potentially augmenting the skills offered by volunteers within such programs. Indirectly, and an outcome arising from their recommendations, veterinarians can contribute to the well-being of the clients served by these programs by helping ensure that well-qualified dog/hander teams work on behalf of canine therapy programs offering support to clients.

## 7. The Clinic Visit—An Informal Assessment Opportunity

It is argued here that the clinic visit provides a rich opportunity for an informal screening by veterinarians of potential dog-handler teams for referral to local canine therapy programs. The visit showcases the handler’s ability to manage the dog in public, maintain control of the dog in the presence of ample distractions (e.g., the waiting room where other dogs/pets may be present), and the introduction of the dog to clinic staff (e.g., for an initial weigh-in by a technician) and to the treating veterinarian. As Fine (2015), in his Handbook on Animal-Assisted Therapy, has argued, observing the dog/handler team in action in a setting where the team encounters an unknown person in an unknown setting is a strong predictor of therapy canine success [29].

In addition to providing insights into the handler’s ability to manage his/her dog, the veterinarian is able to discern the dog’s temperament in a public setting and his or her willingness/interest in meeting new people. In short, the visit provides ample fodder for the veterinarian to make a holistic or general impression of suitability for potential participation in canine therapy initiatives through an informal assessment of dog-handler skills in what could be deemed a stressful situation. Though arguments have been made around reducing stress during clinic visits [13], the clinic visit can be a stressful event for both human and animal clients. Within the context of a clinic visit, the veterinarian is able to see how the dog handles a potentially stressful setting and interaction [30]. The veterinarian is particularly well positioned to discern clients and dogs who stand out given the sheer number of clients a clinician sees in a given day. Recognizing too that the veterinarian is an influential agent in this transaction and his/her suggestion to a client to investigate the possibility of participating in a canine therapy program carries weight. There is thus a greater likelihood of follow-through on the client’s part to seek additional information regarding local therapy programs.

We would be remiss in not addressing the potential risks associated with establishing the partnership described above. Over time, and as a veterinarian becomes linked to, or known for recommending clients to, a local canine therapy program, there is a risk that the clients the clinician recommends to the program do not pass the program’s assessment/evaluation practices. This, in turn, could potentially compromise the veterinarian-client relationship and risk the client seeking services elsewhere. To reduce this risk, the veterinarian must consider: (1) investing time in familiarizing him/herself with the program’s screening, assessment, and selection criteria; and (2) phrasing the recommendation as a suggestion and not an explicit endorsement (e.g., “In light of your dog’s behaviour in the clinic today and how I’ve seen you manage your dog, you might consider looking into volunteer work with a canine therapy program. Let us know if you’re curious to learn more and we can direct you to a local agency.”). An additional risk or complexity lies in larger urban centres having multiple canine therapy programs in operation. Whether to establish an exclusive relationship with one therapy program or to simply provide clients with a list of all programs serving the area, is something for clinicians to decide. A last risk, and one seen more recently within the expanding field of canine therapy, is that some programs are charging an assessment fee, a membership fee, or both. Directing clients to a canine therapy agency that charges an assessment fee, could be misinterpreted by clients as a money-grab, especially if the client and his/her dog are not selected by the program. Despite the above-mentioned risks inherent in community-engagement work of the nature proposed here, there is potential for veterinarians to work in harmony with a local canine therapy program. Next, we propose a stepwise model to illustrate how such a partnership might unfold.

## 8. Proposing a Model for Clinic-Therapy Program Partnership

A review of the extant veterinary and anthrozoological literature revealed no stepwise model to connect a veterinary clinic to a canine therapy program. A model that could successfully connect these two parties is proposed in Table 2. The model proposed below requires minimal time, effort, and resources from both parties in order to create a bidirectional, mutually beneficial partnership.

## 9. Conclusions

Both veterinarians and canine therapy programs have clients or participants with restricted enrolment. That is, despite optimal care, the canine clients of a veterinary clinic will expire and the clinic has a standing need to bring in new clients. This is paralleled by canine therapy programs who see dogs retire due to advanced age or whose handlers move or have life events that prevent participation in programming. Thus, both agencies have a need to replenish their client base and it has been argued here that a collaborative model in which veterinarians informally assess and recommend dog-handler teams for participation in local canine therapy programs and canine programs in turn, help cultivate clientele for the veterinarian, is a model that stands to benefit both parties.

## Figures and Tables

**Table 1:** Professional responsibilities of veterinarians across associations.

Association:	Code of conduct regarding community education:
British Columbia Veterinary Medical Association (BCVMA) [25]	All veterinarians as members of a learned medical profession, owe a duty of service to the public and in fulfilling this duty must maintain the highest standards of integrity and ethical conduct. Members should make efforts to contribute to the education of the public in matter relating to and promoting the health and safety of animals and thereby the public; but members must do so in accordance with generally recognized standards of integrity and professionalism.
Canadian Veterinary Medical Association (CVMA) [26]	A veterinarian should continue to study, apply, and advance scientific knowledge, make relevant information available to clients, colleagues, the public and maintain a commitment to veterinary medical education. The responsibilities of the veterinary profession extend beyond individual patients and clients to society in general. Veterinarians are encouraged to make their knowledge available to their communities and to provide their services for activities that protect public health and environmental health.
American Veterinary Medical Association (AVMA) [27]	A veterinarian shall continue to study, apply, and advance scientific knowledge, maintain a commitment to veterinary medical education, make relevant information available to clients, colleagues, the public, and obtain consultation or referral when indicated. A veterinarian shall recognize a responsibility to participate in activities contributing to the improvement of the community and the betterment of public health. The responsibilities of the veterinary profession extend beyond individual patients and clients to society in general. Veterinarians are encouraged to make their knowledge available to their communities and to provide their services for activities that protect public health.
Federation of Veterinarians of Europe (FVE) [28]	Veterinarians should make animal owners aware of their responsibilities to the public. Veterinarians should, whenever appropriate, advise their customers about measures to minimize the risk of zoonotic agents, food borne pathogens, residues, contaminants (biological and chemical agents) and antimicrobial resistance. Veterinarians may inform the public about their services in an accurate and not misleading manner. Such communication must be truthful, transparent and correct.

**Table 2:** Stepwise model to build clinic—canine therapy partnership.

Therapy Program Personal	Veterinarian/Clinic Staff
1. Therapy program identifies and contacts a potential collaborating local veterinary clinic to book an appointment with a veterinarian.	1. Veterinarian accepts appointment to discuss opportunity.
2. Therapy program attends appointment with documentation and explains the benefits of a partnership.	
3. Therapy program invites veterinarian to observe a new handler/new dog session to observe desired behaviors. Provides explicit instruction to veterinarian on all aspects of screening, assessment, and selection of dog/handler teams.	2. The veterinarian learns the program’s screening, assessment, and selection practices.
4. Therapy program invites veterinarian to attend a therapy session familiarizing them with the program operation and environment.	3. Veterinarian is familiarized with therapy program goals and expectations by attending session.
5. Therapy program enters informal agreement with participating veterinarian clinic.	4. Veterinary clinic enters informal agreement with therapy program.
6. Therapy program endorses veterinary clinic on website or social media and through dog handler referrals.	5. Veterinarian informally screens clients during routine clinical visits for potential recommendation to therapy program.
7. Therapy program maintains regular communication with veterinary clinic about potential therapy candidates.	6. Veterinary clinic maintains regular communication with therapy program about potential therapy candidates.
8. Therapy program follows through on recommendations by veterinarian.	
